# Circulating tumor cells in the early detection of human cancers

**DOI:** 10.7150/ijbs.71768

**Published:** 2022-05-01

**Authors:** Zixin Feng, Junyu Wu, Yuanjun Lu, Yau-Tuen Chan, Cheng Zhang, Di Wang, Dan Luo, Yuan Huang, Yibin Feng, Ning Wang

**Affiliations:** 1 School of Chinese Medicine, The University of Hong Kong, Hong Kong S.A.R, P.R. China; 2 School of Life Science, Jilin University, Jilin, P.R. China; 3 Cellomics International Limited, Hong Kong S.A.R., P.R. China

**Keywords:** Circulating tumor cell (CTC), Early detection, Liquid biopsy, Human cancers, Cancer metastasis

## Abstract

Cancer is a severe disease with high morbidity and mortality globally. Thus, early detection is emerging as an important topic in modern oncology. Although the strategies for early detection have developed rapidly in recent decades, they remain challenging due to the subtle symptoms in the initial stage of the primary tumor. Currently, tumor biomarkers, imaging, and specific screening tests are widely used in various cancer types; however, each method has limitations. The harms are even overweight against the benefits in some cases. Therefore, early detection approaches should be improved urgently. Liquid biopsy, for now, is a convenient and non-invasive way compared to the traditional tissue biopsy in screening and early diagnosis. Circulating tumor cells (CTCs) are vital in liquid biopsy and play a central role in tumor dissemination and metastases. They have promising potential as cancer biomarkers in early detection. This review updates the knowledge of the biology of CTC; it also highlights the CTC enrichment technologies and their applications in the early detection of several human cancers.

## Introduction

Cancers have become one of the most malignant diseases that endanger human health. Over 19.3 million individuals were newly diagnosed with cancer worldwide in 2020, most of them having invasive or metastatic disease 1. Metastasis is a prodigious obstacle to cancer treatment 2. It is well established that the high morbidity and mortality of human cancer are due to the late diagnosis and limited therapies 3.

Early detection of cancer can dramatically reduce the death rate. In most cases, surgical operations are feasible options only if the malignancies are diagnosed early enough. Unfortunately, clinically proven biomarkers that can be used for precise diagnosis and patient management are not always available in the early course of diseases. The tumor markers in serum such as carcinoembryonic antigen (CEA) 4, human chorionic gonadotropin (HCG) 5, prostate-specific antigen (PSA) 6, alpha-fetoprotein (AFP) 7, and cancer antigen-125 (CA-125) 8 are common but of poor accuracy and efficacy, for these proteins sometimes unexpected high in the blood of healthy people. Another common strategy for early detection, imaging-based methods, have shown a certain degree of efficacy in multiple clinical studies for decades, but they have their limitations 9. For example, mammography 10 and low-dose CT (LDCT) 11 are used in breast and lung cancer, respectively. Sometimes they lead to overdiagnosis due to the false-positive rate. Colonoscopy (CSPY) allows for removing precancerous polys, but it has a risk of infection and perforations 12. Other approaches for early detection of cancer, such as sputum cytology, stool-based molecular tests, or pap test, are limited to specific tumor types and challenged by poor compliance of patients 11-13. To sum up, there is currently no effective method to detect multiple human cancers.

Nowadays, the development of liquid biopsy has led to a new generation of clinical utility, especially early detection of cancer. This kind of method is based on fluid phase analysis. Single cells or clusters can detach from the primary tumor site, travel through the bloodstream and reside in distant tissues. These cells are known as circulating tumor cells (CTCs). They represent the characteristics of a certain tumor and act as the seed for metastasis 14. It is worth mentioning that CTCs are not just clones of each other. Although some populations have similar identifications, most represent heterogeneous characteristics at different stages and in different types of malignant tumors. Once they receive specific signals from a changing microenvironment or are stimulated by the therapeutic stress, these CTCs will change their phenotype and molecular properties 15. Therefore, liquid biopsy based on CTC analysis is more feasible and less invasive to evaluate tumor heterogeneity than solid biological tissue biopsy 16. Here, we overview the biology of CTC, introduce several CTC capture technologies, and explore their role in the early detection of different types of human cancers.

## Biology of CTCs in cancer progression

**Figure 1:**
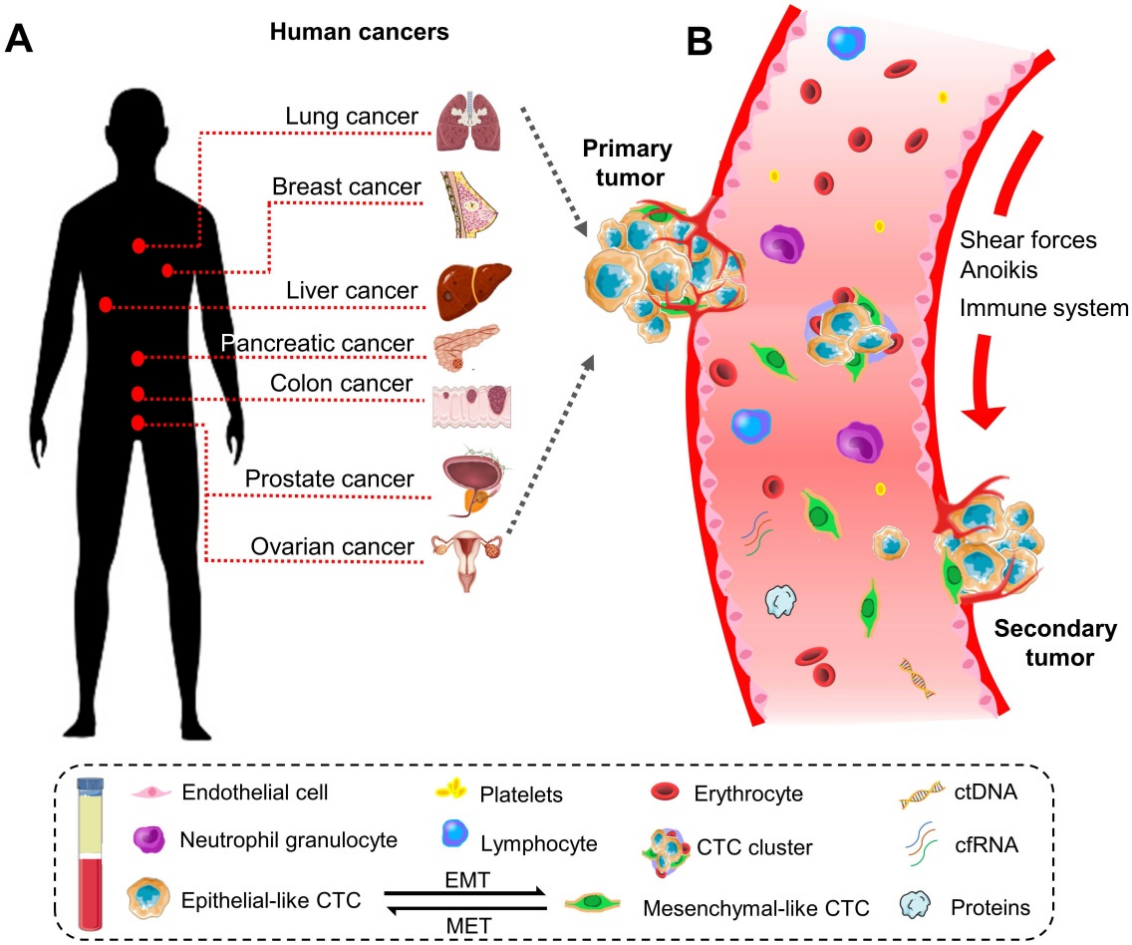
The biology of circulating tumor cells (CTCs) in human cancers. **(A)** CTC is a significant indicator of the disease progression or death in patients with solid metastatic cancer (e.g., lung, breast, liver, pancreatic, colon, prostate, ovarian, *etc*.).** (B)** Schematic representation of CTCs shedding from primary tumor foci, circulating through the blood vessels, and creating a secondary metastasis in the distant organs. In general, CTCs undergo epithelial-to-mesenchymal transmission (EMT), where cancerous epithelial cells lose their cellular connection and gain a more aggressive mesenchymal characteristic. CTCs overcome the stresses in blood flow and reach the distant site. Then they transition back to the epithelial characteristics and develop metastatic tumor masses. CTC can present as a single cell or gather into a cluster to enhance the metastatic ability.

Historically, the initial description of CTCs can be dated back to 1869. A pathologist Thomas Ashworth found a small group of cells in the blood of patients with cancer and considered they were tumor cells originating from the cancerous tissue 17. Indeed, CTCs play a critical role in the metastasis of human cancers 18. They can disseminate from primary sites (e.g., breast, colorectum, ovary, liver, pancreas, prostate, and lung) through the circulatory system to distant parts of the body. A representative diagram is presents in Figure 1.

The high cancer-associated mortality is mainly attributed to the metastatic spread, which involves multiple steps, including tumor cells intravasate into the bloodstream accompanying neovascularization, overcome the pressure in the circulation, extravasate to the new environment, and finalize the colonization to form a secondary tumor mass (Table 1). Because dissemination frequently occurs through the circulatory system, especially in blood, studying the biology of CTCs can elucidate the molecular mechanisms of the metastatic process and provide insights into the cancer progression 19.

### Intravasation and angiogenesis

The first step for CTCs to participate in metastatic cascades is to access the proximal vessels. Interestingly, CTCs can enter the bloodstream in both active and passive ways 20, 21. For the active intravasation, CTCs are motile and migrate directly. They go through the basement membrane and penetrate the endothelial layer of their own accord. To fulfill this mission, CTCs change their morphology and molecular properties to make themselves more aggressive and invasive. This dynamic process is especially essential to cancer metastasis and frequently occurs in embryogenesis and wound healing, termed epithelial-mesenchymal transition (EMT) 22, 23. During EMT, CTCs reprogram their epithelial characteristics to mesenchymal phenotype by reducing epithelial markers like epithelial cell adhesion molecules (EpCAM), cytokeratins (CKs), E-cadherin, and correspondingly upregulating mesenchymal markers such as N-cadherin and vimentin. EMT is induced and regulated via the coordination of intracellular and extracellular actors. When it is triggered by some extracellular factors such as transforming growth factor-beta (TGFβ), epidermal growth factor (EGF), hepatocyte growth factor (HGF), insulin-like growth factor (IGF), and fibroblast growth factor (FGF), it will activate transcription factors including Twist, Snail, Slug, and Zeb to maintain mesenchymal characteristics 24. Consequently, the close-connected and well-organized epithelial cells loosen the tight junctions with each other and lose the interaction with the cellular matrix to gain the ability to detach from the tumor foci. Notably, the activation of EMT will give rise to intermediate cells defined as cancer stem cells (CSCs), which have a state between epithelial and mesenchymal 23.

As for the passive way, CTCs are mobile; they are dragged or pushed into the circulation by external forces 25. Although this kind of way is not well studied by far, the tumor vasculature and microenvironment contribute to the living condition of the CTCs. Remarkably, a significant hallmark of cancer is angiogenesis. Carcinomas induce vascular endothelial growth factor (VEGF) secretion, which facilitates sprouting new vessels from the existing ones, providing necessary oxygen and nutrients 26.

### Survival maintenance

After access to blood circulation, CTCs are exposed to adversity and struggle for survival. Although the underwent EMT tumor cells are more resistant to the shearing force, oxidative stress, and collisions with the other cellular components in blood flow, the vast majority of them are limited due to loss of adherence to a matrix 27. Absence of the essential extracellular matrix proteins, the homeless CTCs that travel in the suspension liquid will induce a programmed cell death process termed anoikis 28. Once this kind of apoptosis triggers, the pro-apoptotic proteins like Bak and Bax would repress the anti-apoptotic counterparts including Bcl-XL, Bcl-W, and Mcl-1. To overcome the challenging condition, CTCs need to defect the death receptor pathway of caspase by activating tropomyosin-related kinase B (TrkB) and the caspase-8 inhibitor FLIP to suppress apoptosis and block the mitochondrial pathway by upregulating the Bcl-2 family 29, 30.

Another obstacle that affects CTCs' survival is the attack by a diversity of immune cells. Even so, CTCs can employ multiple mechanisms to avoid immune surveillance. For instance, upregulating a “don't eat me signal” CD47, can protect CTCs from macrophages and dendric cell killing. Besides, forming a programmed death-1 (PD-1) and its ligand (PD-L1) complex strongly suppresses T-cells. Thus, CTCs that express PD-L1 can prevent destruction by the immune system. Moreover, downregulating Toll-like receptors (TRL) on macrophages and Natural killer (NK) cells also potentially helps damage tumor surveillance 31. In addition, forming CTC clusters and shortening the stay time in the bloodstream contribute to increasing CTCs survival. Therefore, CTCs are rare (generally no more than 10 cells in 10 mL of blood) and have a short half-life time (less than 10 min for cluster and half an hour for single cells).

### Extravasation and colonization

CTCs that survive in the bloodstream finally dock in branch points between vessels or tiny capillaries, and then extravasate through the walls of the endothelium to outgrow metastasis in the foreign microenvironment 32. However, the mesenchymal-like CTCs are motile but not conducive to growth 33. It is therefore CTCs need to undergo a reversal process of EMT, known as mesenchymal-epithelial transition (MET), to regain their epithelial characteristics. During MET, epithelial markers of CTCs are upregulating while mesenchymal properties are repressed. Indeed, CTCs are highly competent for epithelial-mesenchymal plasticity 34, 35. Beyond that, primary cancer and direct organ release certain cytokines to upregulate the E-selectin on endothelial and facilitate CTCs to penetrate vessel walls 36. After coming out from the bloodstream, CTCs cross the basement membrane, stay in the stoma, and grow locally to form a secondary tumor.

Upon the extravasation, CTCs present gene expression heterogeneity in different types of human cancer. For example, CTCs in breast cancer can be characterized by the expression of Her2, Notch1, EGFR, and HSPE, but the absence of EpCAM. This population of tumor cells prefers to develop brain metastases 37. Also, EGFR ligand HBEGF and COX2 were two regulators identified in metastatic models that selectively metastasize to the brain 38. It suggests that analyzing the gene expression pattern on CTCs can evaluate the destination of metastatic spread.

Among the extravasated CTCs, a subpopulation migrates to the bone marrow and lingers in a dormant state for a long time until metastases become evident, which can last for decades. These tumor cells are called disseminated tumor cells (DTCs) 39. Many types of malignant diseases such as breast, lung, and colorectal cancer are likely to occur in bone metastases. Thus, bone marrow is considered the reservoir for DTCs 40, 41. The basis of dormancy may be the initial EMT stage, which prevents DTCs from being traced and waits for the right time to resume proliferation induced by a certain stimulus 42, 43.

## Methodologies for CTC isolation and enrichment

**Figure 2:**
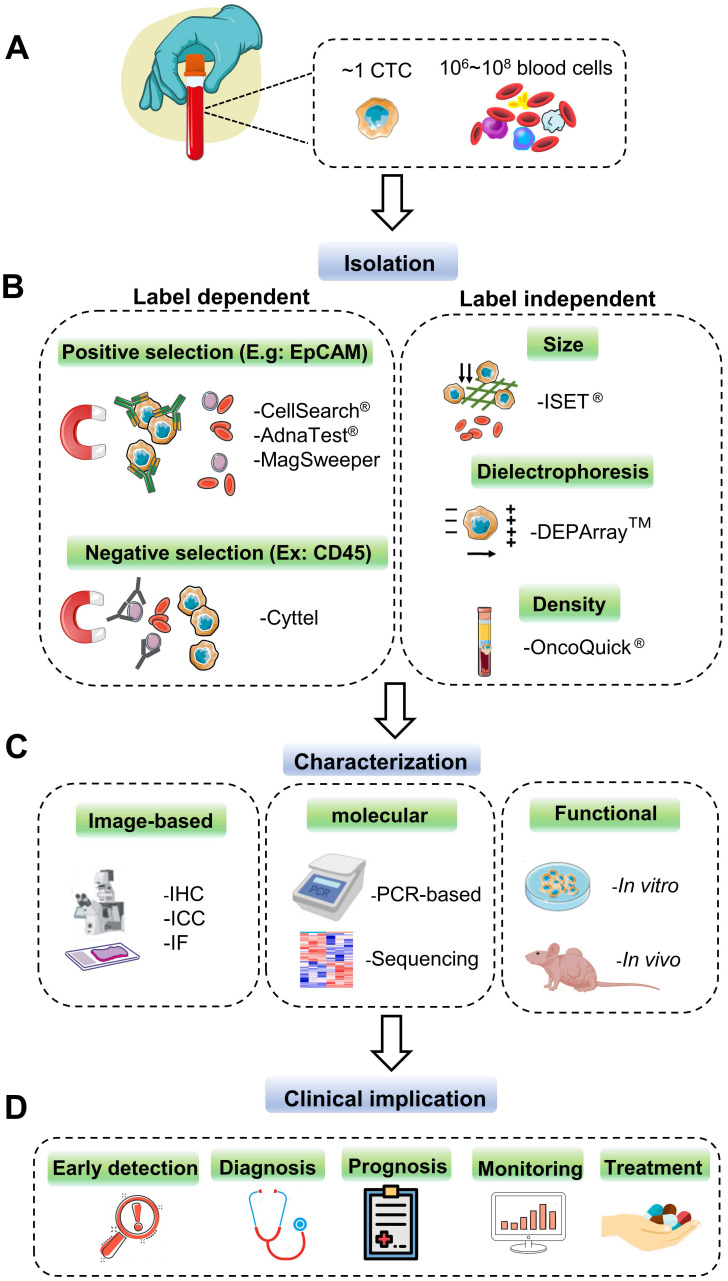
Current technologies for CTC detection. **(A)** CTCs rarely appear in the peripheral blood of cancer patients. Approximately one CTC can be detected per milliliter of the blood sample with millions of background blood cells. **(B)** The strategies for CTCs isolating and enrichment can be divided into label-dependent and -independent techniques, which are based on their biological or physical properties. Among label-dependent techniques, immunomagnetic separation is the most common. EpCAM (epithelial cell adhesion molecule) antibodies can be coated on ferrofluids (CellSearch®) and magnetic beads (AdnaTest® & MagSweeper) for positive selection, while negative selection depletes white blood cell (WBC) by recognizing CD45 (Cyttel). Label-independent methods include enrichment by size (microfluidic chips), electric charge (DEPArray™), and density (OncoQick®). **(C)** After enrichment, the captured CTCs are prepared for downstream analysis by immunostaining using antibodies against cancer-related proteins; by PCR targeting tumor-specific nucleic acid sequences; by *in vitro* cell culture or *in vivo* xenograft model for the following functional research. **(D)** CTCs as liquid biopsy materials have great potential to participate in early detection, diagnosis, monitoring for cancer recurrence, and prediction of individualized treatment.

As the minimal number and heterogeneity of CTCs present in peripheral blood (Figure 2A), powerful and robust methods for selecting and capturing CTCs are very challenging and demanding, which can provide the premise and foundation for the following analysis and further characterization. To achieve this goal, many commercialized CTC capture technologies have been developed in the last decade; each one has its distinctive characteristic for sensitive CTC identification. In this part, we generally classify these techniques into label-dependent and -independent according to their cell surface markers (Figure 2B).

### Label-dependent technologies

The Label-dependent technologies utilize the principle of immunoaffinity to identify and collect CTCs by their specific surface makers that differ from blood cells (Table 2).

#### Positive selection

This approach is the most common system of CTC isolation. By using antibody-conjugated magnetic microbeads, CTC can be identified by immunorecognition of cancerous-related markers, which do not express in leukocytes 44, 45. More specifically, epithelial markers such as EpCAM and CKs are present in CTCs at various levels but absent from white blood cells (WBCs), while CD45 is the opposite 46.

CellSearch® is the first and the only system validated by U.S. Food and Drug Administration (FDA) for CTC measurement in metastatic cancers 47, which is considered the gold standard by far. This approach employs ferrofluid nanoparticles coated with epithelial markers to enrich EpCAM positive CTCs and confirmed by immunostaining with CK8, 18, and 19 without CD45 expression 48. The main advantages of CellSearch® are efficiency and high reproducibility. However, this system has its limitations. It entirely depends on the expression of epithelial markers without considering other potential biomarkers. In fact, EpCAM becomes dramatically downregulated when CTCs undergo EMT to gain the mesenchymal characteristics and are ready for metastasis 49. Another drawback is that CTCs isolated by CellSearch would not be available for the downstream molecular analysis because of the preservative inhibition. Moreover, the CTC purity yield from CellSearch cannot achieve satisfactory results 50.

AdnaTest® is another positive enrichment system that sorts CTCs by immunomagnetic beads. The beads are linked with a series of antibodies to enhance the capturing. AdnaTest® has been extensively used in a variety of human cancers, including breast, ovarian, prostate, and colon cancer 51. qRT-PCR targeting specific genes analyzed the captured cells based on tumor type. Although this approach has better heterogeneity characterization of CTCs, the CellSearch system is considered superior to AdnaTest, especially in metastatic breast cancer clinical studies 52.

In addition to the above two systems, MagSweeper is also a positive section example. It is worth mentioning that Magnetic-activated Cell Sorting (MACS) systems and CTC-iChip devices offer both positive and negative selection for detecting CTCs 53.

#### Negative selection

This kind of isolation is based on the depletion of hemocytes without considering the expression of surface markers on CTCs. The whole procedure is summarized into two steps. Firstly, red blood cells (RBCs) are lysed for the next step. Secondly, the WBC markers, such as CD45 and CD61 are used to magnetically deplete the leukocytes from the sample 54-56. A classic negative selection system, RosetteSep™ uses both RBC and WBC markers to double target the erythrocytes and leukocytes and remove most of them from the peripheral blood sample, which entirely relies on non-cancerous blood cells and is independent of the cellular surface markers of CTCs. RosetteSep™ has a higher recovery rate than the traditional density gradient approach 57. Cyttel is another negative immunomagnetic selection method, which depletes WBCs by recognizing CD45, then performs gradient centrifugation to collect CTCs. This approach has a high detection rate with bimodal identification of CTCs and is applied in the prognosis of lung cancer 58.

### Label independent technologies

Given that CTCs express epithelial markers like EpCAM and CKs to variable degrees in different cancer stages, some isolation platforms based on the biophysical properties have emerged as the alternative choice. These strategies recognize CTCs by their size, density, and electrical properties (table 3).

#### Size-based

Currently, several size-based isolation systems have been well established, including microfluidic chips, membrane filters, and hydrodynamic approaches. These systems isolate the bigger CTCs from other smaller cellular ingredients without relying on the tumor cell surface markers 59, 60. Microfluidic chip is a regular size-based CTC selecting technique termed “three-dimensional microfiltration”, with specific stereoscopic spaces for the classified tumor and non-tumor cells. The Parsortix system is such an example, and this platform is formed into a trapezoid shape and gradually narrows to capture the cells of interest. This construction prevents the target cells from being deformed. CTCs are usually bigger than the channel gaps thus are trapped, while smaller peripheral blood cells can pass and be removed. The instrument lengthens the separation channel as much as possible and allows the counterflow. These conditions make the downstream CTC analysis available 61. It is pretty easy and reliable to isolate CTCs by size filtration, but the main pitfall is the low recovery efficiency due to the accumulation of filtration resistance.

A breakthrough technology called Cellomics' CTC cell sorting platform has been developed in recent years. The platform also employs microfluidics chips, which select CTCs by their size, shape, and morphology, with 4mL of blood sample in no more than 5 minutes, and the accuracy rate is over 90%.

#### Density-based

Density gradient centrifugation is a typical segregation approach for separating different compositions according to their density. As blood is in the liquid phase, cells are distributed along the gradient depending on their sedimentation coefficients. RBCs and WBCs with heavier cellular density precipitate at the bottom, whereas tumor cells and platelets remain at the top 62. The OncoQuick^®^ system is technologically upgraded by using a porous membrane to avoid mingling up with each constituent. OncoQuick^®^ is simple and inexpensive. Unfortunately, the yield rate is not desirable.

#### Electric charge-based

Dielectrophoresis (DEP) can select the cells of interest with an exceptional level of accuracy, which is a method to exploit electrical properties between different cells, depending on cell composition, morphology, and phenotype 63. The DEPArray™ system is the latest technology for the precise isolation of rare cells from heterogeneous samples. It is designed with 320 × 320 arrayed electrodes, enabling every CTC to correctly guide into the individual spherical cage. This system aims perform excellently in single CTC detection 64.

## Strategies for CTC Identification and characterization

After isolation and collection, various technologies can be used to characterize and analyze CTCs to provide essential insights into the following cancer therapeutic strategies (Figure 2C). These technologies are achieved by image-based approaches, including immunocytochemistry (ICC), digital image capture and analysis, and nucleic acid analysis like Real-Time Quantitative Polymerase Chain Reaction (RT-qPCR), multiplex RT-qPCR, and tumor-related protein identification 21, 65.

Morphologic investigation with ICC by using antibodies against CK is commonly applied for the qualitative and quantitative analysis of CTCs after gathering. However, the identification of CTCs by conventional immunofluorescence, typically when it is manually observed and judged by skilled technicians, is labor-intensive and time-wasting. Another automated choice that can make up for these defects is conducted by the laser scanning cytometer of super-efficiency, which can screen high-enriched CTCs. Nevertheless, the main drawback is its high non-specific binding. Ariol high-throughput automated image analysis system is also widely used for imaging CTCs. This system satisfies the quality requirements of diagnostic pathology images at high resolution without gathering the proteins released by CTC and evaluated in different types of cancer, like lung, colorectal, and prostate 66-68.

PCR-based assays are more sensitive and specific. For example, RT-qPCR assays can target specific genes of tumor cells only but not in non-cancerous hemocytes, at the concentration of one CTC in over 10^6^ leukocytes 69. This method extracts the total RNA from CTCs and employs the RT-PCR to amplify the tumor-related gene sequences 65. For instance, cancer-associated markers (EpCAM, mucin1, and ERBB2); EMT-related transcription factors Twist1, Snail, phosphatidylinositol 3-kinase alpha (PI3Ka), Akt-2, as well as stem cell markers CD34, CD133, aldehyde dehydrogenase 1 (ALDH1) are some target genes 70.

## Clinical implications of CTCs in human cancers

The following part focuses on the clinical value of CTC (figure 2D) and current technologies used to select and collect CTCs in various human cancers. Also, the pros and cons of traditional early detection approaches and CTC-based methods applied in different carcinomas will be discussed.

### Breast cancer (BC)

According to World Health Organization (WHO), breast cancer (BC) is the most prevalent cancer in females, with more than 2.3 million new cases diagnosed in 2020 1. BC has a strong metastasis capability and usually transfers to the other organs, which mainly accounts for its incurability. Early detection can result in a favorable outcome. In some developed countries, the survival rates of BC patients are greatly improved due to timely treatment 71.

Screening or diagnosis of BC by using radiographic imaging has been applied for decades, like mammography, ultrasonography, X-ray, positron emission tomography (PET), and Magnetic resonance imaging (MRI) 72-75. However, it can only examine a small part of the tumor tissue, which provides limited information on tumor characteristics. Besides, these imaging methods cannot access the tumor; thus, they do not provide a good understanding of tumor biology. Therefore, isolation of the liquid biopsy components circulating in the bloodstream or other cancer-derived materials in body fluids for further analysis has become an attractive alternative strategy 76.

CTC is a dynamic prognostic biomarker and the main component of liquid biopsy, which has been collected for large datasets in BC patients through a variety of CTC detection methods in the past decade. CTC has been studied the most in BC than in the other types of cancer. The first challenge of CTC-based diagnosis is to develop a suitable platform in a cost-effective and time-saving way.

In 2004, a study firstly reported the significant clinical validity of CTC count in metastatic breast cancer (MBC) by CellSearch® system, showing that the survival of BC patients can be predicted in advance, and it depends on how many CTCs are detected 77. Since then, CellSearch has been the most frequently used CTC isolating and enrichment system. It mainly depends on capturing the EpCAM positive cells. Nonetheless, only 70% of MBC patients can detect CTCs by this method, let alone in non-metastatic settings 78. Later in 2007, a rapid and sensitive CTC detection method for BC patients was developed. This study used nucleases, which are elevated in EMT-induced BCs, as CTC biomarkers for signal amplification. The results showed that fluorescent nuclease-activated probes could rapidly examine CTC levels as a method of early detection in a quick, inexpensive, and easy way 79, 80. A decade since then, more and more strategies have been developed for CTC detection from the blood of BC patients, such as AdnaTest®, Magsweeper, MACS, CTC-Chip, and DEPArray™. However, CellSearch® is still the only FDA-approved platform for detecting and analyzing CTC.

Up to now, studying CTC is a promising approach for BC early detection, but its improvement is highly desired, and further research should focus on CTC detection methods and their utilization in cancer diagnosis 79.

### Colorectal cancer (CRC)

The incidence of colorectal cancer (CRC) is rising every year, along with the high death rate. CRC is always detected at its late stage, which leads to unfavorable results. However, it can prevent in some way 81. Screening provides an opportunity for early detection of cancer. Currently, there are several options for Colorectal cancer (CRC) screening that can improve the survival rate, such as CSPY, fecal immunochemical tests (FITs), guaiac-based fecal occult blood tests (FOBTs). These stool-based tests are validated by the U.S. Preventive Services Taskforce. However, global CRC screening rates are meager. The reasons are complex, including the reluctance of patients, high cost, and time-consuming 82, 83. For effective screening, an acceptable test should exist to detect the early stage of CRC.

Tissue biopsies have been extensively reviewed and used in early detection for many years, but there are many risks during invasive operations, including bleeding, visceral perforation, and infections. Sometimes this traditional method causes anguish to the patients when the tumor is difficult to reach in a sensitive part of the anatomy. Additionally, the tumor is heterogeneous, and the solid tissue sample would be restricted spatially and temporally. Therefore, tissue biopsies are not always feasible 84.

Over the past few years, liquid biopsy has taken over the lead of traditional biopsy. More cancer information can be obtained in the blood sample by CTC analysis. CTC can be the prognosis marker in CRC. In non-metastatic cancers, patients without detectable CTCs have better outcomes, and lower or absent CTCs is independent predictor of non-disease and non-metastasis survival. According to prospective research, the median progression-free survival (PFS) and overall survival (OS) are much longer in CRC patients with a favorable CTCs baseline compared to those who have an unfavorable CTC baseline. These findings suggested that the CTC level in baseline and follow-up provide a strong prediction for the survival rate 84, 85. The commonly used approaches for isolating CTCs from patients with CRC are primarily based on immunomagnetic selection; AdnaTest® and MagSweeper are the frequent choices. Subsequently, the CTCs identification is measured by PCR-based methods or sequence analysis.

Until now, CellSearch® is not only the gold standard for CTC detecting in MBC but is also approved in CRC 86. Despite some advances, a remaining challenge is the low detection rate. Therefore, recent approaches aim to massively augment the analyzed blood volume by using diagnostic leukapheresis (DLA) or functionalized catheters for *in vivo* CTC capturing 87.

### Ovarian cancer (OC)

Epithelial ovarian cancer (EOC) is the main type of ovarian cancer (OC), which is usually found at an advanced stage and leads to gynecological cancer death 88. Generally, when the primary foci confine to the ovary, 90% of OC patients can receive adequate treatment. However, there is only 25% of cases can be detected before exacerbating 89. Therefore, early detection of OC remains an important goal.

Currently, CA-125 is a common serum biomarker for diagnosing OC in the early stage. It is a high molecular weight glycoprotein, which presents in the body fluid of EOC patients and can be tested by immunoassays 90, 91. Besides, more than 100 biomarkers combined with CA-125 show better effects in detecting the early course of OC than either alone. Among all, the most promising candidate is human epididymis protein (HE4). When these two tumor markers are together to predict the malignancy, the sensitivity and specificity can reach 76.4% and 95%, respectively 92.

There is no doubt that the combination of CA-125 and HE4 is the efficient tool to diagnose OC, while CTC was demonstrated more sensitive in predicting progressive disease. The functional CTC isolation and enrichment can be conducted by cell adhesion matrix, and the number of CTC is closely correlated with PFS and OS, which is more accurate than protein tumor markers 93. To further characterize the CTCs in OC, the most widely used method is molecular assays. Six specific genes that target the female cancers are analyzed by RT-qPCR, showing that CTCs are detected in 19% of ovarian cancers, 44% of cervical, 64% of endometrial cancer 94. Another study using MetaCell to evaluate the cytomorphology and molecular characteristics of CTCs by fluorescence microscopy and gene expression analysis. It is confirmed that the expression of EpCAM, CA-125, MUC1, KRT7, KRT18, KRT19 in OC patients are significant differences. Therefore, they show promise to become the helpful markers involved in the early detection and predict the therapeutic response of OC 95.

In addition, CTC has a significant prognostic value in various types of human cancer and proved to be a better choice for monitoring OC than common serum biomarkers. Taken together, CTC may exhibit more potential in the detection of OC, especially in the early course of the disease 93, 96.

### Hepatocellular carcinoma (HCC)

Hepatocellular carcinoma (HCC) is a typical type of liver cancer, accounting for 75~85% of the total. It exhibits significant aggressiveness at the advanced stage and results in a poor prognosis; thus, no curative therapy is available 1.

Alpha-fetoprotein (AFP), the most widely used serum biomarker for HCC, has limited sensitivity for early detection 7, 97. Besides, imaging-based methods show a certain degree of efficacy in multiple studies but also expose their shortcomings, as they can only provide partial information on tumor characteristics. Even the combined testing of AFP and ultrasonography is far from satisfactory due to insufficient efficacy and accuracy. Apart from these conventional approaches, liver biopsy allows direct sampling of tissue that can reveal the biology of the tumor. However, it is not used routinely because of its invasiveness and tendency to tumor seeding, especially in the early course of the disease 98. So, there is an urgent need to develop an HCC-specific and sensitive biomarker to improve the cancer screening and early detection of HCC.

CTC has been proved to be involved in the spread of cancer as a critical metastatic initiator, thus considered the new biomarker of early detection of HCC 99. Numerous studies show that the early detection of HCC mainly relies on CTC evaluation, which is also associated with cancer staging, tumor metastasis, and the AFP level in the blood of patients 100. CTCs can be sorted and classified from the fluid sample by applying immunomagnetic isolating techniques and *in situ* hybridization. With further enumeration and characteristics, CTC shows its sensitivity in recognizing the HCC. More than 90% of HCC patients were CTC positive, even in the early course of the disease 101. In a recent study, CTCs were also found in the body fluid of patients with hepatitis B virus (HBV) infection, and these CTC-positive patients actually developed an HCC tumor within a few months 101. Moreover, CTCs also implicated in cancer staging 102. Its number is a positive correlation with tumor node metastasis (TNM) staging, which may assist in classifying the TNM staging and improve the diagnostic efficiency.

Nowadays, the CTC with epithelial phenotype has emerged to investigate in HCC. Although the knowledge about the clinical correlation between CTC and HCC is less intensive than the other cancer types 103, the survival rate of HCC is significantly associated with CTC 102, 104. So, it is clear that the CTCs may serve as a real-time parameter for measuring the HCC 105. However, some research indicated that CTC alone might not be ideal for predicting the HCC. Combining AFP and CTCs would improve the sensitivity of early detection 99, 106.

### Pancreatic cancer

Pancreatic cancer is one of the deadliest cancer types, with a patient survival rate among the worst of any solid cancer. Among all the pancreatic cancers, pancreatic ductal adenocarcinoma (PDAC) is the majority of all cases. This devastating malignancy usually leads to an abysmal prognosis. So delayed diagnosis is the main reason for death 107, 108. Generally, the symptom is subtle in the initial stage of pancreatic cancer, and presents clinically silent. Once the disease becomes increasingly apparent, tumor cells have invaded the adjacent tissue or even migrated to the distant organs. Early diagnosis and tumor resection is the most effective therapy for pancreatic cancer and the greatest hope for patients 109.

Up to now, liquid biopsy is the minimal harm method and direct means to indicate the progression of PDAC, except the pancreatic juice collection, of course. It is well known that CTC is the indicator of disease progression in pancreatic cancer 110, 111, which can be shed from primary lesions like intraductal papillary-mucinous neoplasia (IPMN) and pancreatic intraepithelial lesions (PanIN) early in PDAC development. The expression of EMT-related genes in CTCs reveals that some of them are derived from PanIN, while the expression of epithelial-associated genes and noncoding RNA (HSATII) indicates that the other of them come from IPMN 112, 113. After immunomagnetic enrichment of the CTC in PDAC, molecular characteristics can be conducted to analyze the expression of PDAC specific genes. E-cadherin and Muc-1 are showing a decrease, while Cadherin11, SPARC, and Aldh1 are upregulating 114.

Overall, it has been practically guaranteed that CTCs have a certain use value as a biomarker in PDAC. However, there are still some barriers that stand in the way of screening and early detection in pancreatic cancer, such as insensitivity of detection, rarity, and heterogeneity of CTC. Indeed, the technologies of CTC detection are in an immature stage, leading to varying results. In order to satisfy the requirement of large-scale clinical application in pancreatic cancer, improved methodologies for CTC detecting should be established 115-117.

### Prostate cancer

Prostate cancer is the second most frequent cancer that threatens the health of males worldwide 1. The prominent feature of prostate cancer is bone metastasis, but the tumor limited to the prostate may be curable. Androgen deprivation therapy remains the cornerstone of treatment for recurrent or metastatic disease. Unfortunately, nearly all patients will develop resistance to androgen blockade leading to castration-resistant prostate cancer (CRPC) 118.

In the early course of prostate cancer, the decreased number of CTC is a response to posttreatment 119. It can also help decide on therapy as an indicator 120. Recent studies of CRPC are trying to incorporate the detection of CTCs, imaging-based methods, and patient information to improve the management and drug development 121. Previously, some researchers have valued the quantification of CTC in metastasis prostate cancer, which considers it has the effective implementation of prognosis 122, before or after the treatment 123. Soon after, the FDA-approved CellSearch system for capturing and collecting CTCs in CRPC showed that the enumeration of CTC could predict the OS of the CRPC patients 124. Afterward, many studies have further confirmed that the CTC enumeration can also monitor the disease status 125, 126. Besides, CTC has a positive correlation of PSA level and TNM staging in metastatic hormone-sensitive prostate cancer, which can correctly stage the disease progression 127, 128. According to a prospective phase III study, the CTC baseline can aid the prognosis and optimize the therapy 129.

Although PSA testing, the standard screening of prostate cancer, increases the detection rate, there is controversy over whether it improves outcomes. Besides, the value of PSA, a prostate cancer-specific maker, for early detection needs to be refined 130. To date, liquid biopsy-based early detection has gained considerable attention. According to some studies, CTC assays are not sensitive enough to detect prostate cancer 131. But combining CTC with other components in liquid biopsies, such as circulating tumor DNA (ctDNA), microRNA, and exosomes may overcome the limitations.

### Lung cancer

Lung cancer is the first malignancy with the highest morbidity and mortality. Non-small cell lung cancer (NSCLC) takes a large portion of the total, accounting for about 80%. Small cell lung cancer (SCLC) is the second most common type of lung cancer, comprising 15% of all cases 132. Only the NSCLC is identified at an early stage and receives a timely surgical resection would the patients have a good prognosis 11, 133. Unfortunately, more than 75% of NSCLC is diagnosed at advanced 134. SCLC is more aggressive than NSCLC, which means a worse prognosis. Once SCLC is detected, most of them already have local or distant dissemination 134. Although the window for treatment is narrow, surgical resection can be a benefit to some extent 135. Therefore, it is absolutely reasonable to develop an effective early detection biomarker in NSCLC and SCLC 11.

CTC is a powerful tool for monitoring the progression of malignancies, especially when the lung biopsy causes uncomfortable for the patients 136. There is considerable evidence that CTC analysis can help screen the malignancy in patients with pulmonary nodules 137. Moreover, combining the CTC screening with immunohistochemistry or gene expression profiling can identify the origins of primary foci. The tumor cells that express TTF-1 and KRT7 come from NSCLC 137. Recently, a study confirmed that pulmonary vein (PV) blood sampling combined with a sensitive microfluidic chip capture system could provide a much higher yield of CTCs in early-stage lung cancers than reported previously 138. Differences in CTCs levels were identified at the preoperative, intraoperative, and postoperative intervals, with a decrease postoperatively. Most importantly, the number of CTCs is associated with the tumor size 138, 139. Nevertheless, the value of CTC in the early detection of lung cancer should be studied more, alone or combined with other tests, in order to avoid a false-positive diagnosis. The over-diagnosis can be further verified by LDCT screening 140.

Given the aggressive characteristics of SCLC, the diagnosis should be performed timely when detected the CTCs in the blood of the patient, even in the initial stage of the disease 141. Therefore, CTC is a promising candidate for early detection and cancer screening. In general, people with benign masses or healthy ones can hardly detect CTCs 142, while patients with SCLC have a more significant number of CTC 143, 144. According to some studies, both CTC and ctDNA are in the experimental stage in a clinic, but they show potential in evaluating the biology of tumors 145-147. Moreover, comparing CTC with the cells in circulating tumor microemboli (CTM) by analyzing the molecular characteristics may provide novel insights into SCLC 143, 148.

## Discussion

Tissue biopsy as a conventional strategy in the early detection of cancers can only provide partial information on the tumor or hardly be obtained in some cases. Actually, the snapshot of the sample and non-specific findings are variable in the immunohistochemical analysis, which may lead to misdiagnosis and ineffective therapy 149. Furthermore, cancer is a highly metastatic and recurrent disease, which means patients may have a risk of metastasis when removing solid tissue for diagnosis. Besides, patients must undergo routine monitoring and a series of follow-ups even after surgery. The multiple biopsy procedures will accompany surgical complications and much pain in the course of treatment. To this end, more and more efforts are being made to satisfy the need for effective and efficient early detection and screening tests 16, 150.

A new non-invasive diagnosis tool called liquid biopsy can obtain comprehensive information on heterogeneous carcinomas. In a liquid biopsy, tumor-derived fragmented nucleic acids including ctDNA and cell-free RNA (cfRNA), soluble proteins, metabolites, and cells are the measured parameters of cancer that can be analyzed in the human blood. As for the tumor-related proteins, like CEA, AFP, CA19-9, PSA, HCG, and CA-125 are used to detect colorectal, liver, pancreas, prostate, and ovarian cancers, respectively. However, these protein biomarkers are not intact cells, while CTCs can present the cellular phenotype 151. In addition, compared to another liquid biopsy component ctDNA, CTC can reflect the different ingredients of the tumor, not just a fragment of circulating nucleic acid from an apoptotic tumor cell 151. Taken together, CTCs show more promising potentials for multiple screening than tissue biopsy and other components in liquid biopsy.

In spite of significant advances that have been made in diverse CTC isolation and enrichment systems and trying to be applied in clinical implementation, some hurdles impede the development of CTC-based early detection tests. The main obstacle is that multiple types of cancer cannot be identified without knowing the subtle symptoms or specific mutations in advance 152. Another major barrier is the inaccuracy of detection. Even CellSearch, which is the only successful testing platform that obtained FAD approval, indicates limited sensitivity. When CTCs are assayed utilizing CellSearch, half of the advanced cancer could not be able to be detected, and the detectable rate has no significant difference between people without cancer and patients in benign conditions or early stages of malignancy 9, 153. Furthermore, it is difficult to compare the output between different systems in cancer screening, diagnosis, and prognosis because of the substantial variation. To date, a standardized approach for CTC enrichment and characterization is still lacking in clinical. The instabilities and uncertainties of the CTC detection results may influence the interpretations and decisions of the diagnosis and therapeutic regimen. Since the clinical relevance of CTCs is still unclear, it is difficult for any CTC detection technology to be introduced into routine clinical practice in the short term 53.

On the other hand, CTC studies would be relatively time-consuming and high cost considering the expense. CTCs are rare, which means that more than 7 mL blood draws are needed. Accordingly, patient compliances would be reduced when they need to phlebotomize frequently, particularly in the situation of continuous follow-up 65. Additionally, due to the tumor heterogeneity and cell recovery, CTCs are not easy to absolute count and may miss some information. Thus, more efforts should be made in improving the methods to promise greater yield and increase accuracy.

## Conclusions

There is no doubt that CTC exerts great promising functions as an early detection tool in different human cancers that do not have a perfect screening method. In the last decade, along with better understanding of CTC and its role as a biomarker in liquid biopsy, a myriad of technologies was developed aiming to apply in clinical practice. However, limited sensitivity and specificity are the reason for the challenges of early detection assays. When combined with the other components of liquid biopsy, traditional imaging, or tumor protein biomarkers, CTCs can hold the substantial potential to be a pivotal player in the screening tests, which may give more comprehensive information about the patient and predict the most suitable cancer therapy. In summary, CTC has opened up a new avenue of early detection but still has a long way to go.

## Figures and Tables

**Table 1:** Mechanism of CTCs participate in cancer metastasis

Biological process of cancer metastasis	Characteristics of CTCs	Expression of the related markers on CTCs	Expression of the factors in tumor microenvironment	Outcomes
Intravasation and Angiogenesis	- Motility (active intravasation); - Mobility (passive intravasation); -Epithelial to Mesenchymal plasticity	EpCAM, CK8/18/19, E-cadherin↓; N-cadherin, Vimentin, Fibronectin↑; Snail, Slug, Zeb, and Twist1↑; N-WASP↑ (Invadopodia formation)	TGF-β↑ (CAF); VEGF, PDGF↑ (platelet); VEGFA, FGF↑ (macrophage);	-CTCs undergo EMT and enter into bloodstream; -New vessels formation
Survival maintenance	-Anoikis resistance; -Rarity; -Short half-life time; -Single cells or clusters	CD47, PD-L1, FASL↑; Bcl-2 family, TrkB, FLIB↑; Bax, Bak↓	TLRs↓ (macrophage and NK cell); CD41, CD61↑ (platelet)	-CTCs overcome the shear stress and anoikis; -CTCs escape from the immune surveillance
Extravasation and Colonization	-Mesenchymal to Epithelial plasticity; -Homing and dormancy; -Heterogeneity	EpCAM, CK8/18/19, E-cadherin↑; N-cadherin, Vimentin, Fibronectin↓; TGF-β,VEGF, MUC1, CD44, Integrins, CXCR4, CXCR7↑	TGF-β↑ (platelet) E-selectin↑ (endothelial cell); CXCL12↑ (stroma);	-CTCs undergo MET and leave the bloodstream; -Outgrowth of metastasis or remain dormant

**Table 2:** Technologies for CTC detection and their application in human cancers (Label-dependent)

Technologies	Isolation and enrichment	Identification and characterization	Specification and performance	Limitations	Merits	Cancer type	Ref.
-CellSearch^®^ system	-Positive selection; -EpCAM-coated ferrofluid nanoparticles for positive selection of CTCs	-The captured cells are confirmed by IF stained with CK 8, 18,19 but an absence of CD45	-Sensitivity: 27%, 32%, 70%; -Specificity: 89%, 99.7%, 93%; -Recovery: 80%	-Miss EMT-CTCs due to EpCAM dependent	-First FDA approved, -Most clinical validated capture technique	-Breast, -Colorectal, -Prostate	52, 154
-AdnaTest^®^	-Positive selection; -Using a cocktail of antibodies to enhance the enrichment	-Tested by multiplex RT-PCR for various gene panels (e.g., prostate: KLK3, PSMA, and EGFR; breast: MUC-1, Her2)	-Sensitivity: 73%; -Positive rate: 40% (88 of 221)	-Contamination with WBCs	-High sensitivity; -Can analyzes bone marrow sample	-Breast, -Colon, -Ovarian, -Prostate	51, 52, 155, 156
-MagSweeper	-The magnetically-labeled CD133+ cells were purified	-Whole transcriptome analysis with RNA-Seq	-Sensitivity: 100% -9mL/hr -Detect 1-3 CTCs /mL	-Expensive; -Unable to further analysis due to CTCs being fixed or lysed	-High purity; -High throughput processing	-Breast, -Prostate, -Colorectal	157, 158
MACS system	-Both positive and negative selection; -Immunomagnetic isolation with anti-pan CK antibody	-Automated analysis after combined anti- CK/CD45/DAPI staining	-Detect rate: 5/17	-Identifies EpCAM negative cells but not CK negative ones	-High efficiency	-Lung (NSCL), -Breast, -Pancreatic	159
CTC-iChip	-Both positive and negative selection; -Deterministic lateral displacement, inertial focusing, and magnetophoresis	-Molecularly characterized by RT-PCR	-Detection limit: <30 CTCs/7.5 mL; -Processes: 8mL/h; -Recovery: 98.6%; -Throughput:1-2 ml/h	-Low purity (around 8%); -Complicated fabrication; -Potential RBC contamination	-Combination of biological and physical properties	-Prostate	160, 161
RosetteSep™	-Negative selection; -Using tetrameric antibody complexes that recognize WBC and RBC (CD45, CD66b, and glycophorin)	-Followed by flow cytometry	-Sensitivity: 59%; -Specificity: 87%; -Diagnosis accuracy: 75%	-Low recovery rate	-Sensitive	-Pancreatic, -Breast, -Colorectal	53, 162
Cyttel	-Negative selection; - followed by gradient centrifugation	-Slide smearing; -In situ hybridization	-Sensitivity: 83.05%; -specificity:100%	-Lack of standardized clinical protocols	-High detection rate; -Bimodal identification;	-Lung (NSCLC), -Colorectal	58, 163

**Table 3:** Technologies for CTC detection and their application in human cancers (Label-independent)

Technologies	Isolation and enrichment	Identification and characterization	Specification and performance	Limitations	Merits	Cancer type	Ref.
Parsortix™ system	-Size (employ Microfluidic chips)	-Immunostaining (CD45, CK, and vimentin)	-Capture rate: 81% -Sensitivity: 92%; -Specificity: 100%	-Buffy-coat enrichment and CTC loss due to different size ranges	- Allows reverse flow	-Ovarian; -Prostate	53, 61, 164
ISET^®^	-Size (filter-based isolation and enrichment)	-IHC	-Sensitivity: 76.37% -Specificity: 82.39%	-Miss CTCs due to their morphological heterogeneity	-High efficiency compared to CellSearch	-HCC, -Lung	165, 166
OncoQuick^®^	-Density (separation of blood cells by porous membrane filtration then followed by density-grade centrifugation for CTC collection)	-RT-PCR targeting CEA, CK20, and TEM-8 in CRC;	-Recovery: 87%; -Throughput: 7.5mL/40min	-Low purity; -Miss large CTC or cell aggregates; -Additional techniques are needed	-Price reasonable; -Reliable	-Colorectal; -Metastatic BC;	167
DEPArray™	-Electric charge based for single CTC capture	-Next-Generation Sequencing (NGS)	-N/A	-Limited volume; -Electrical properties of CTCs may be affected; -Operation complex	-Single CTC isolation; -High cell viability	-Early stage of BC;	168, 169

## Abbreviations

AFP: alpha-fetoprotein
ALDH1: aldehyde dehydrogenase 1
AR: androgen receptor
BC: breast cancer
CA-125: cancer antigen-125
CEA: carcinoembryonic antigen
cfRNA: cell free RNA
CK: cytokeratin
CRC: colorectal cancer
CRPC: castration-resistant prostate cancer
CSC: cancer stem cell
CSPY: colonoscopy
CTC: circulating tumor cell
ctDNA: circulating tumor DNA
CTM: circulating tumor microemboli
DEP: dielectrophoresis
DLA: diagnostic leukapheresis
EGF: epidermal growth factor
EMT: epithelial-mesenchymal transition
EOC: epithelial ovarian cancer
EpCAM: epithelial cell adhesion molecules
FDA: Food and Drug Administration
FGF: fibroblast growth factor
FIT: fecal immunochemical test
FOBT: fecal occult blood tests
HBV: hepatitis B virus
HCC: hepatocellular carcinoma
HCG: human chorionic gonadotropin
HE4: human epididymis protein 4
HGF: hepatocyte growth factor
IGF: insulin-like growth factor
IPMN: intraductal papillary-mucinous neoplasia
LDCT: low-dose computed tomography
MACS: magnetic-activated cell sorting
MBC: metastatic breast cancer
MET: mesenchymal-epithelial transition
MRI: magnetic resonance imaging
MUC16: membrane-spanning mucin
NK: natural killer
NSCLC: non-small cell lung cancer
OC: ovarian cancer
OS: overall survival
PanIN: pancreatic intraepithelial lesions
PDAC: pancreatic ductal adenocarcinoma
PD-1: programmed death-1
PD‑L1: programmed cell death 1 ligand 1
PET: positron emission tomography
PFS: progression-free survival
PI3Ka: phosphatidylinositol 3-kinase alpha
PSA: prostate-specific antigen
PV: pulmonary vein
RBC: red blood cell
RT-qPCR: real time quantitative polymerase chain reaction
SCLC: small cell lung cancer
TGFβ: transforming growth factor beta
TrkB: tropomyosin-related kinase B
TRL: toll-like receptors
VEGF: vascular endothelial growth factor
WBC: while blood cell
WHO: world health organization
